# Microbiome-Guided Exploration of the Microbial Assemblage of the Exotic Beverage “Insect Tea” Native to Southwestern China

**DOI:** 10.3389/fmicb.2019.03087

**Published:** 2020-01-29

**Authors:** Xin Mao, Peter Kusstatscher, Haoxi Li, Xiaoyulong Chen, Gabriele Berg, Maofa Yang, Tomislav Cernava

**Affiliations:** ^1^College of Forestry, Guizhou University, Guiyang, China; ^2^Institute of Entomology, Guizhou University, Guiyang, China; ^3^Guizhou Provincial Key Laboratory for Agricultural Pest Management of the Mountainous Region, Guiyang, China; ^4^Institute of Environmental Biotechnology, Graz University of Technology, Graz, Austria; ^5^College of Tobacco Science, Guizhou University, Guiyang, China

**Keywords:** insect tea, tea microbiome, food microbiome, *Pyralis farinalis*, *Enterobacteriaceae*, Chong Cha

## Abstract

Insect tea is a unique beverage that is native to Southwestern China and traditionally produced by local farmers in an elaborate process. It consists of insect larvae excrements that are commonly obtained from meal moths (*Pyralis farinalis* Linnaeus 1758) reared on a specific plant-based diet. We have reconstructed the whole production process under laboratory conditions in order to obtain microbiome-level insights into this uncommon beverage and to trace back the origin of the prevalent bacteria in the final product. The bacterial community composition was specific for each production stage, with a high proportion of *Streptomycetacea*, *Pseudonocaridaceae*, *Enterococcaceae*, and *Enterobacteriaceae* in the insect tea. A large proportion of the constituents was traced back to the producing insect (13.2%) and its excrements (43.8%), while the initial plant-based substrate for tea production was found to contribute only 0.6% of the traceable bacteria in the final product. Moreover, an enrichment of *Enterobactericeae* was observed during the analyzed process steps and verified with complementary analyses. The cultivation experiments indicated a high occurrence of viable bacteria in the tea at 2.7 × 10^5^ ± 1.2 × 10^5^ cfu g^–1^. The isolated bacteria included *Bordetella petrii* and *Enterococcus* spp. that were recovered from a commercial product. By implementing an integrative approach, the insect tea was shown to harbor a species-rich bacterial community that can be traced back to certain plant and insect microbiome constituents from distinct production steps. Moreover, the microbial profile of the insect tea was found to be unique for a food product so far and contained several bacterial groups that are considered from the current perspective as food contaminants or yet unreported in other beverages. Due to the high number of viable bacteria, the tea harbors a so far undescribed dynamic component that might have implications for human health.

## Introduction

The so-called insect tea (Chinese: 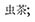; pinyin: Chong Cha) is a traditional beverage of the ethnic minorities in Southern China and mainly produced in the mountainous region south of the Changjiang river (Xu et al., 2013). This tea is a highly exceptional beverage because it solely consists of excrements obtained from insect larvae that were reared on a specific plant leaf diet (Bai, 2010). Nowadays, various commercial products are available in the Chinese market, yet it is a costly niche product that is only consumed by a small group of the population. The traditional procedure to obtain this unconventional product starts with the collection of fresh leaves which are then air-dried and stacked in baskets made of bamboo or other wooden containers. Various plant species can be used for the production of insect tea, including *Cyclocarya paliurus* (wheel wingnut, Juglandaceae) (Wen and Guo, 1997; Xiang and Lu, 1998; Wang et al., 2010). In analogy to the broad substrate range, many insect species from the order Lepidoptera can be utilized to produce insect tea (Qin, 1983; Bai, 2010; Liu et al., 2014). Interestingly, *Pyralis farinalis*, which is commonly known as the meal moth and famous as cosmopolitan pest of stored grain, is also often used for insect tea production (Shang et al., 2011). After rearing the larvae on leaf stacks for several months, their excrements are collected and roasted before they are packed and marketed (Bai, 2010). The quality grade of the insect tea depends on particle diameter, leaf harvesting time, and processing methods; the microbiological parameters are currently not assessed.

Similar to other eukaryotic organisms, insects are colonized by microbial communities of varying complexity that primarily occur in the insect’s gut (Engel and Moran, 2013) and the exoskeleton (Skovgaard et al., 2015); however, some specifically adapted endophytes can also colonize other internal tissues (Eleftherianos et al., 2013). Recent findings related to the insect gut microbiome have shown that it generally harbors a lower microbial diversity as compared to the mammalian guts (Engel and Moran, 2013). The most abundant phyla in various insect species were shown to be *Proteobacteria* and *Firmicutes* (Alonso-Pernas et al., 2017; Kaczmarczyk et al., 2018; van Schooten et al., 2018). Similar to other organisms, the insect-associated microbial communities generally show differences across the life stages of their hosts (Alonso-Pernas et al., 2017; Kaczmarczyk et al., 2018). Moreover, various microbes were shown to be involved in insect growth and development (Shukla et al., 2018). The transmission of the microbiome among insect individuals can be horizontal (Qi et al., 2018) as well as vertical (McManus et al., 2018). In terms of other possible transmission paths, the insect gut microbiome was so far not assessed in connection with its contribution to a food product. While the morphological, biological, and ecological characteristics of meal moths were investigated systematically (Shang et al., 2011, 2013b), their gut microbiome remained uncharacterized. Studies addressing insect tea are rather scarce and focus on its nutritional value and classic microbiological analyses. Recent evaluations of its composition and a biosafety assessment were conducted with products based on *Litsea coreana* and *P. farinalis* larvae (Shang et al., 2013a; Wang et al., 2017). The microbial constituents as well as the contamination of food products are still mostly assessed with standardized cultivation-dependent methods. However, there are various applications that benefit from more detailed community-level assessments of prevailing microorganisms. Examples include the monitoring of fermentation processes (De Filippis et al., 2017), the profiling of microbial communities in ripened products (Quigley et al., 2012), and the linking of product properties with the presence of specific microorganisms (Lattanzi et al., 2013). Due to the broad applicability of microbiome studies in food microbiology, they will likely contribute to the improvement of various food products, which is not feasible with the conventional methods (De Filippis et al., 2018). The pioneering studies addressing the microbial constituents of edible insects with next-generation sequencing delivered community-level insights that will serve as a basis to improve this sustainable protein source in the future (Garofalo et al., 2017, 2019; Vandeweyer et al., 2018). We expected that the insect tea would have a complex bacterial profile due to its origin and the traditional processing steps. Therefore, we implemented it as a model in a systematic approach to show how potential contaminants can be tracked during a production process by using microbiome-based approaches. The objectives of this study were to (1) provide a complete bacterial profile of a representative product, (2) link the plant leaf and insect gut microbiome with the bacteria present in the insect tea, and (3) track selected target bacteria along the whole production process. In a complementary approach, the aerobic mesophilic bacterial fraction of commercial products was cultivated in order to evaluate if they contain living microorganisms that could potentially have implications for human health. *P. farinalis* (insect) and *C. paliurus* (substrate for the plant-based diet of the larvae) were implemented in all of the experiments conducted because they are most commonly used for insect tea production in Southwestern China. The obtained results provide a first insight into the bacterial community composition of a unique product and a basis to further explore its active ingredients.

## Materials and Methods

### Larvae Rearing and Sample Preparation

The *P. farinalis* larvae were obtained from a large colony maintained at the Institute of Entomology, Guizhou University, Guiyang, China. Only larvae that are commonly used for the insect tea production (approximately fourth to sixth instar) were selected for the experiments. Transparent plastic boxes (11 × 11 × 9 cm) were sterilized with 75% ethanol and then placed under a UV lamp (Taisite Instrumental Company; Tianjin, China) for 12 h. Small holes on the top of the containers ensured sufficient aeration during the insect rearing. Six replications were prepared under the same conditions. Each of the containers was equipped with five *C. paliurus* leaves and 20 larvae from the initial colony. The containers were kept at 22°C, RH 80%, and 14-h/10-h day/night cycles for 5 days in a growth chamber (Jiangnan Instrumental Company, Ningbo, Zhejiang, China). This initial step was included for acclimatization and renewal of the gut content. Then, the larvae from each container were transferred into new containers with the same amount of fresh *C. paliurus* leaves. The leaf samples for the subsequent DNA extractions were obtained from these containers before the larvae were added. After another 7 days, the excrements were collected separately from each container. At the same time, the midguts from the larvae were separated with sterile forceps and scalpels under a dissecting microscope. All insect larvae were freeze-killed at -20°C according to current research and industry standards before the midgut was removed (Larouche et al., 2019). All samples were stored on ice during processing and subsequently used for the total community DNA extractions. A commercial insect tea product (Chishui Chong Cha, Chishui, Guizhou, China) that is produced by the same insect larvae species and a plant-based diet that is not strictly defined was implemented for comparative analyses. The product was stored at room temperature in sealed sachets before it was transferred to extraction tubes under sterile conditions.

### Total Community DNA Extractions

All samples were processed with a DNA extraction kit (FastDNA SPIN Kit for Soil; MP Biomedicals, Solon, OH, United States) to extract the total community DNA. Leaf, excrement, midgut, as well as commercial insect tea product (Chishui Chong Cha, Chishui, Guizhou, China) samples were directly transferred into the extraction vials provided in the aforementioned kit in order to avoid contaminations. The sample types and the experiment procedures are schematically shown in Supplementary Figure S1. For the leaf and excrement samples, approximately 150 mg was used for each replicate. The insect midgut samples were pooled for each replicate. They were obtained from 13 larvae reared in the same container, with a total weight of approximately 150 mg for each of the six samples. The samples of the commercial product (155 mg per replicate) were obtained from separate sachets. The total community DNA extractions were conducted according to the manufacturers’ protocol, and the extraction efficiency was confirmed by photometric determinations of the DNA concentration (NanoDrop 2000, Thermo Fisher Scientific, Wilmington, DE, United States). For each sample type, six biological replicates were obtained. The total community DNA extracts were stored at -20°C until further processing.

### Barcoding and High-Throughput Sequencing of 16S rRNA Gene Fragment Amplicons

The aforementioned DNA samples were transferred to a sequencing company (Novogene Co., Ltd., Beijing, China) for next-generation sequencing that targeted the 16S rDNA Hypervariable Region 4 (V4). The following PCR program (95°C for 5 min to denature the DNA, 30 cycles at 96°C for 60 s, 78°C for 5 s, 54°C for 60 s, 74°C for 60 s, and 10 min at 72°C for a final extension) was used for the generation of amplicons according to the standardized protocol provided by the Earth Microbiome Project^1^. During the PCR amplification, 1 μl of mPNA and pPNA were added to all samples in order to block the amplification of plastid and mitochondrial 16S rDNA sequences (Lundberg et al., 2013). The PCR blockers were obtained from PNA Bio Inc. (Newbury Park, CA, United States). In addition, bovine serum albumin (BBI Solutions, Shanghai, China) was used to increase the efficiency of the PCR reactions. The next-generation sequencing was conducted by Novogene (Beijing, China) on the Illumina PE250 platform that produces 2 × 250-bp paired-end reads.

### Bioinformatic Analyses

The reads were assigned to samples by their unique barcode sequences and the barcode which were truncated by the sequencing company (Novogene Co., Ltd., Beijing, China). Demultiplexed paired-end reads were imported into QIIME 2 (2019.1 release; Caporaso et al., 2010), and the DADA2 algorithm (Callahan et al., 2016) was applied to summarize sequence variants (SVs) and to generate a filtered feature table as well as representative sequences. The chimeras were filtered from the table, and the features were classified using a Naïve–Bayes classifier trained on the SILVA 132 release (Quast et al., 2013), with 99% similarity and a confidence level of 0.7 (default value). The mitochondria and the chloroplasts were further excluded from the table, resulting in a total of 3,260,703 reads which are assigned to 8,465 features. The determination of alpha and beta diversity was performed using the QIIME 2 core diversity metrics and group significance tests. The dataset was rarefied to a depth of 42,300 reads per sample. The feature table was split into four tables according to the sample group (leaf, midgut, excrement, and insect tea). Subsequently, the core microbiome was generated by filtering the features present in four out of the six samples (66.6%). All core microbiomes were unified again, resulting in a total of 1,082 core features that were retained. The table was exported from QIIME 2, and barplots were generated with a cutoff of 1% abundance. For network rendering, the features with less than 1,000 reads were further filtered from the core feature table (296 features retained) and collapsed on the genus level. Using the make_out_network.py script in QIIME 1.9.1, this collapsed feature table was exported for visualization in Cytoscape 3.6.1 (Shannon et al., 2003). The SourceTracker 0.9.5 software embedded in QIIME was used to predict the source of the microbial communities in the different sample types.

### Verification of *Enterobacteriaceae* Enrichment in the Samples

In order to confirm the accumulation of *Enterobacteriaceae* during the production process of the insect tea, a qPCR-based approach was employed. The same DNA extracts that were used for the amplicon sequencing of the 16S rRNA gene fragments were adjusted to a concentration of 2 ng/μl in order to account for the differences in the extraction efficiency. The primers *rplP* 1F (5′-ATG TTA CAA CCA AAG CGT ACA-3′) and *rplP* 185R (5′-TTA CCY TGA CGC TTA ACT GC-3′) were used according to the method described by Takahashi et al. (2017) to quantify the *Enterobacteriaceae*-specific markers. All quantifications were performed with the PowerUp SYBR Green Master Mix (Applied Biosystems; Vilnius, Lithuania) and the CFX96 Real-Time System (Bio-Rad Laboratories, Hercules, CA, United States). Each sample was analyzed with six technical replicates and two independent runs. The mean fluorescence intensity of the leaf samples was used as a reference to determine increases in fluorescence intensity during the production process as well as in the final product.

### Isolation and Quantification of Aerobic Mesophilic Bacteria in Commercial Insect Tea

In order to isolate bacteria from a commercial insect tea product (Chishui Chong Cha, Chishui, Guizhou, China), a total of 40 tea sachets, containing 3 g of insect tea each, from at least two production batches were homogenized in 30 ml 0.85% NaCl on a shaker (Jinghong Co., Ltd., Shanghai, China) at 120 rpm for 10 min. Subsequently, a dilution series (1:10 steps) was prepared and plated in triplicate on LB agar (non-selective complex growth medium; Saigon Biotech Co., Ltd., Shanghai, China), MacConkey agar (selective medium for Gram-negative bacteria; Lianmai Bioengineering Co., Ltd., Shanghai, China), and chromogenic coliform agar (selective medium for coliform bacteria; Hope Bio-Technology Co., Ltd., Qingdao, China). The plates were incubated at 30°C and used for the cfu determination after 48 h (only LB plates) and for the isolation of bacterial cultures (all plates) during a period of 7 days with daily inspection of the plates. For the isolation approach, the bacterial colonies were selected according to unique morphological features from all of the implemented cultivation media that included visible colonies. The colonies were purified by streaking and sub-cultivation on LB agar. The taxonomic identity of the 14 isolated bacteria (11 from LB agar and three from MacConkey agar) was assessed by DNA extraction and the subsequent amplification of a 16S rRNA gene fragment with the primer pair 27F and 1492r (Lane, 1991). The fragments were analyzed with Sanger sequencing (Saigon Biotech Co., Ltd., Shanghai, China) and aligned with entries in NCBI’s nucleotide database^2^ using the BLASTn tool.

### Statistical Analyses

The statistical tests within the microbiome datasets were performed in the QIIME 2 and the QIIME 1.9.1 software packages. The significance of differences in the alpha diversity was tested with the implemented Kruskal–Wallis test and in the beta diversity with the anosim test in the QIIME 2 pipeline. The differential occurrences of specific features in the different sample groups were statistically verified with the Kruskal–Wallis test with Bonferroni correction (*p* < 0.05) in QIIME 1.9.1 (group_significane.py script). The statistical analysis of the qPCR data was conducted with the non-parametric Mann–Whitney *U*-test.

### Availability of Data and Material

The datasets used and/or analyzed during the current study are available in the ENA repository^3^ under the accession number PRJEB32315. The 16S rRNA gene sequences used for the identification of bacterial isolates were deposited at GenBank^4^ under the accession numbers MK796111–MK796124.

## Results

### Assessment of Bacterial Diversity and Composition Among Different Samples

**FIGURE 1:**
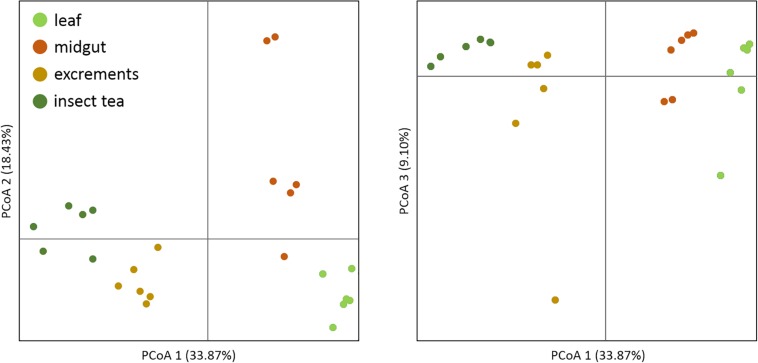
Beta diversity assessment of different sample types. A principal coordinate analysis was conducted in order to visualize the clustering and similarity of the analyzed sample types. The three dimensions which explain the highest degree of variance were included in the visualization. Different colors of the dots indicate distinct sample types. The significance of dissimilarity between the different bacterial communities was tested with anosim in QIIME 2.

For an overall characterization of the bacterial communities, the alpha diversity was calculated for each of the sample types. The Shannon index (*H*) was used to compare the bacterial diversity among the different samples: insect tea (*H* = 8.0 ± 0.6) as well as larvae excrements (*H* = 8.0 ± 0.5) had the highest alpha diversity. In contrast, the alpha diversity in the leaf (*H* = 6.6 ± 0.2) and in the midgut (*H* = 5.1 ± 0.6) samples was statistically significantly lower (*p* < 0.05). The beta diversity assessments showed that the bacterial community composition was significantly different (*p* < 0.05) in each of the analyzed sample types. When a principal coordinate analysis was conducted, a close clustering of the insect tea and the excrement samples was observed (Figure 1).

**FIGURE 2:**
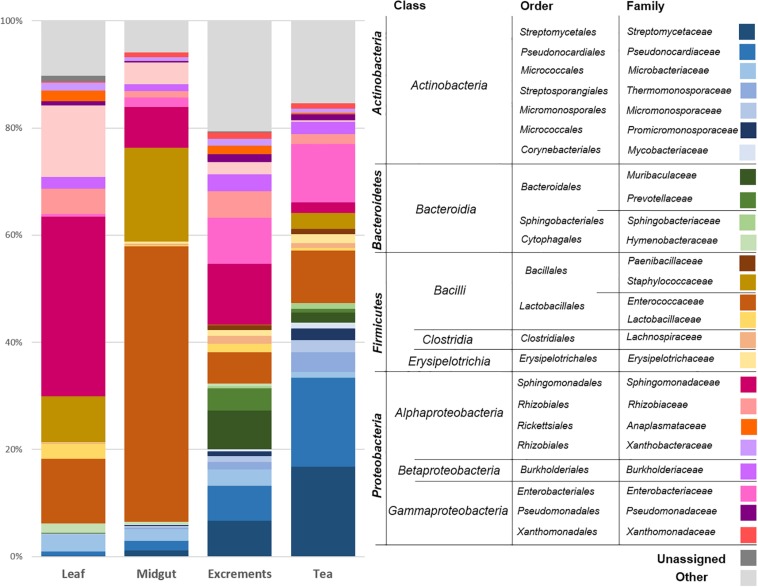
Bacterial community structure of the different sample types. The bacterial community structure was visualized up to family level for each sample type. Taxonomic assignments at higher levels are provided and clustered in the legend. The average abundance of each taxon in six replicates for each sample type (leaf: *C. paliurus* leaves, midgut: *P. farinalis* midguts, excrements: *P. farinalis* excrements, tea: commercial insect tea product) is shown. All taxa with an occurrence of ≥1% were included in the legend. Taxa with a lower abundance were summarized as “other.”

The leaves of *C. paliurus* were primarily colonized by *Proteobacteria* (average abundance 66.2%), followed by *Firmicutes* (22.1%) and *Actinobacteria* (5.7%). *Bacteroidetes* (2.3%) and a group of unassigned bacteria (1.5%) were less abundant. The taxonomic assignments at the class level showed a predominance of *Alphaproteobacteria* (61.2%), *Bacilli* (21.8%), *Actinobacteria* (class, 5.5%), and *Gammaproteobacteria* (4.6%). The prevalent bacterial orders were identified as *Sphingomonadales* (35.3%), *Rhizobiales* (21.4%), *Lactobacillales* (13.5%), *Bacillales* (8.2%), and *Micrococcales* (3.1%). On the family level (Figure 2), *Sphingomonadaceae* (35.3%), *Beijerinckiaceae* (13.8%), *Enterococcaceae* (11.5%), *Staphylococcaceae* (8.0%), *Rhizobiaceae* (5.4%), and *Microbacteriaceae* (3.0%) were the prevalent taxa. *Sphingomonas* (32.5%), *Methylobacterium* (12.9%), *Enterococcus* (11.5%), *Staphylococcus* (8.0%), and *Aureimonas* (3.0%) were the most common genera in the leaf samples.

The midguts of the *P. farinalis* larvae were mainly colonized by the phyla *Firmicutes* (67.4%), *Proteobacteria* (20.8%), and *Actinobacteria* (9.4%). *Bacteroidetes* (0.9%) and other phyla accounted only for a minor proportion of the community. The prevalent bacterial classes were identified as *Bacilli* (66.4%), *Alphaproteobacteria* (16.4%), *Actinobacteria* (9.2%), and *Gammaproteobacteria* (4.2%). *Bacteroidia* (0.9%) and *Clostridia* (0.6%) were less abundant. On the order level, *Lactobacillales* (44.2%), followed by *Bacillales* (22.2%), *Sphingomonadales* (8.8%), and *Rhizobiales* (7.1%), was the most abundant representative. *Micrococcales* (3.8%), *Pseudonocardiales* (2.1%), *Enterobacteriales* (1.5%), and *Clostridiales* (0.6%) were less abundant. On the family level, *Enterococcaceae* (43.9%), *Staphylococcaceae* (21.8%), *Sphingomonadaceae* (8.8%), and *Beijerinckiaceae* (4.7%) were the prevalent taxonomic groups. *Enterococcus* (44.0%) was the predominant genus in the midgut samples, followed by *Staphylococcus* (21.8%) and *Sphingomonas* (8.0%). The genera *Methylobacterium* (4.4%), *Streptomyces* (1.3%), as well as two genera not further classified but belonging to *Pseudonocardiaceae* (1.0%), and *Enterobacteriaceae* (0.9%) were less prevalent.

The excrement samples produced under laboratory conditions and the commercial tea had a similar bacterial community composition; however, differences in the abundance of the most prevalent groups were observed. The prevalent phylum was either *Proteobacteria* (excrements [*e*] = 39.0%, tea [*t*] = 22.8%) or *Actinobacteria* (*e* = 22.4%, *t* = 50.1%) followed by *Firmicutes* (*e* = 13.4%, *t* = 20.3%) and *Bacteroidetes* (*e* = 20.2%, *t* = 5.2%). On the class level, high abundances of *Actinobacteria* (*e* = 21.9%; *t* = 49.4%), *Alphaproteobacteria* (*e* = 22.8%; *t* = 6.5%), *Bacilli* (*e* = 9.9%; *t* = 17.2%), *Gammaproteobacteria* (*e* = 15.8%; *t* = 16.0%), and *Bacteroidia* (*e* = 20.2%; *t* = 5.2%) were observed. The prevalent bacterial classes were represented by *Bacteroidales* (*e* = 18.7%; *t* = 3.3%), *Lactobacillales* (*e* = 8.2%; *t* = 12.4%), *Streptomycetales* (*e* = 6.2%; *t* = 16.9%), *Pseudonocardiales* (*e* = 5.8%; *t* = 15.7%), *Sphingomonadales* (*e* = 11.0%; *t* = 2.1%), *Enterobacteriales* (*e* = 8.0%; *t* = 10.1%), *Rhizobiales* (*e* = 8.9%; *t* = 3.3%), and *Micrococcales* (*e* = 4.8%; *t* = 6.6%). On the family level, *Streptomycetacea* (*e* = 6.2%; *t* = 16.9%), *Pseudonocaridaceae* (*e* = 5.8%; *t* = 15.7%), *Enterococcaceae* (*e* = 5.4%; *t* = 11.2%), *Sphingomonadacea* (*e* = 11.0%; *t* = 2.1%), *Enterobacteriaceae* (*e* = 8.0%; *t* = 10.1%), *Muribaculaceae* (*e* = 10.4%; *t* = 1.9%), and *Prevotellaceae* (*e* = 6.3%; *t* = 0.6%) were prevalent. The most abundant genera were assigned to *Streptomyces* (*e* = 5.9%; *t* = 16.7%), *Enterococcus* (*e* = 5.4%; *t* = 11.2%), and *Sphingomonas* (*e* = 9.9%; *t* = 1.6%). Two abundant representatives of *Enterobacteriacea* (*e* = 6.2%; *t* = 9.2%) and *Pseudonocardiaceae* (*e* = 3.5%; *t* = 10.5%) remained unassigned at the genus level.

### Common Occurrence of Distinct Taxa in the Insect Tea Production Process

**FIGURE 3:**
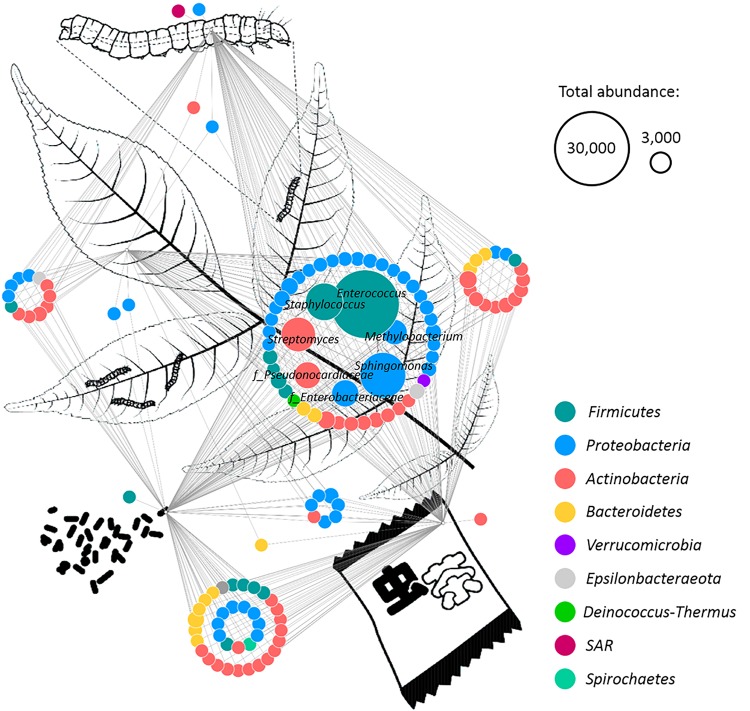
Identification of common microbial components in different sample types. A network was rendered with Cytoscape 3.6.1 in order to identify shared bacterial taxa among different samples. The different sample types (plant leaves, insect midgut, excrements, and insect tea) are schematically visualized. Edges connect each feature with its origin, while multiple edges indicate occurrence in different sample types. The predominant features were labeled with their taxonomic assignment at family (indicated with “f”) or genus level. Node colors indicate the assignment to distinct bacterial phyla, and their size correlates with the abundance of each feature. Two representative node sizes are shown in the legend.

A network based on the core microbiomes of each sample type was generated in order to visualize bacterial genera that are either specific for one of the production steps or such that are shared by at least two sample types (Figure 3). The taxonomic features shared by all sample types were primarily identified as members of the phyla *Proteobacteria* and *Firmicutes.* In total, 48 features were present in all sample types, including *Enterococcus*, *Sphingomonas*, *Staphylococcus*, *Streptomyces*, *Enterobacteriaceae*, *Pseudonocardiaceae*, *Methylobacterium*, *Phylobacterium*, *Saccharopolyspora*, *Lactobacillus*, *Aureimonas*, and *Pseudomonas*. Moreover, 25 of those core features occurred with a total abundance of at least 10,000 reads. The samples from the excrements and the tea shared the highest number of features. They shared 36 taxonomic groups that were not present in the other two sample types. These features included 14 members of *Actinobacteria* and eight members of *Proteobacteria*. The bacterial phylum *Spirochetes* was only present in these two groups and not present in the core microbiome shared by all sample types. The midgut, the excrement, and the tea samples shared 16 features, while the midgut, the leaf, and the excrement samples shared 12 unique bacterial genera. The leaves, the excrements, and the insect tea had seven distinct features in common that were not present in the midgut. Only four genera were found to be unique for one of the sample types. A member of *Betaproteobacteria* and an unidentified SV with the closest match to the eukaryotic SAR (Stramenopiles, Alveolata, Rhizaria) supergroup were only found in the larvae midguts, while bacteria from the class *Erysipelotrichia* were only present in the larvae excrements, and the genus *Phycicoccus* was only found in the commercial tea product.

### Origin Tracking and Sample-Specific Accumulations of Bacterial Groups

**FIGURE 4:**
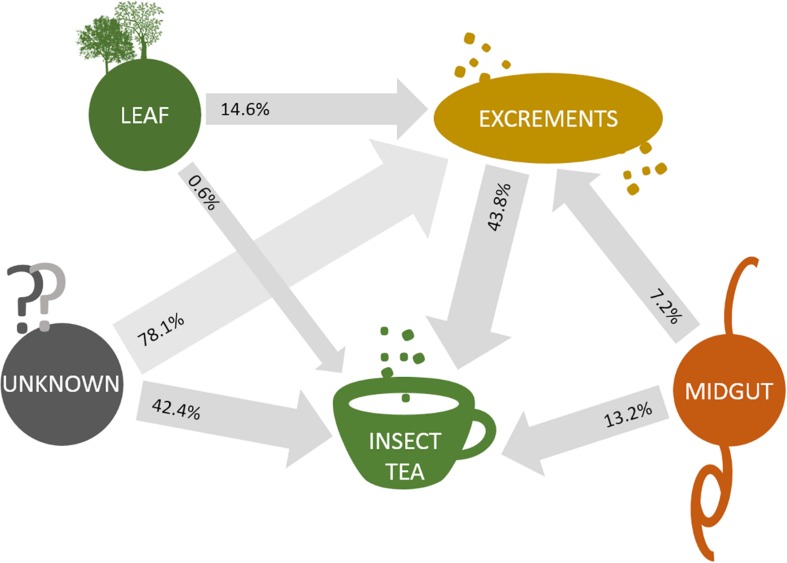
Source tracking of bacterial constituents in insect tea. SourceTracker 0.9.5 was employed in order to identify the source of the bacterial communities that are transferred between different sample types. Transfer routes are visualized with arrows, and the percentage of assignable transferred constituents is provided for each sample pair that was analyzed. Routes were exclusively constructed to follow the direction of the production steps.

**FIGURE 5:**
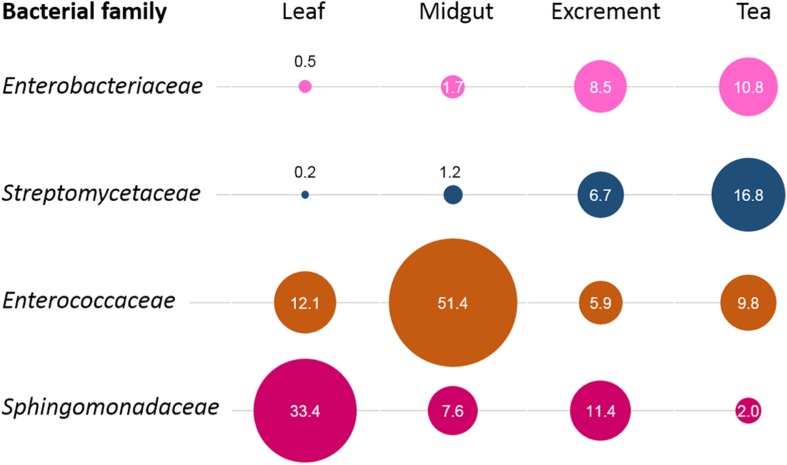
Process-step-specific enrichments of distinct bacterial families. For each process step, the enrichment of distinct bacterial families was observed. The identified families were unevenly distributed among sample types and showed a clear predominance at a distinct production step. The bubble sizes correlate with their relative abundance in the respective sample type, while the numbers indicate their exact percentage.

When the origin of the microbial communities was tracked at different production steps, it was shown that the leaf microbiome of *C. paliurus* contributes only to a minor part of the bacterial composition of the insect tea (Figure 4). While the microbial community of the larvae excrements produced under controlled conditions still contained 14.6% constituents that can be tracked back to the leaves, the commercial product only contained 0.6%. When source tracking was applied to the insect tea and the larvae excrements produced under laboratory conditions, 43.8% of the bacterial population was assignable. The bacteria originating from the larvae midgut were better established in the insect tea (13.2%) than in the excrement samples (7.2%). When the prevalent bacterial families were followed through the production process, each stage showed a distinctive accumulation of a specific lineage (Figure 5). The insect tea samples had the highest prevalence of *Enterobacteriaceae* (10.8%) and *Streptomycetaceae* (16.8%), which were both shown to gradually increase during the production process. The leaf and the midgut samples were characterized by *Sphingomonadacea* (33.4%) and *Enterococcaceae* (51.4%), respectively, which decreased during the subsequent production steps.

### Complementary Molecular and Cultivation-Dependent Assessments

**FIGURE 6:**
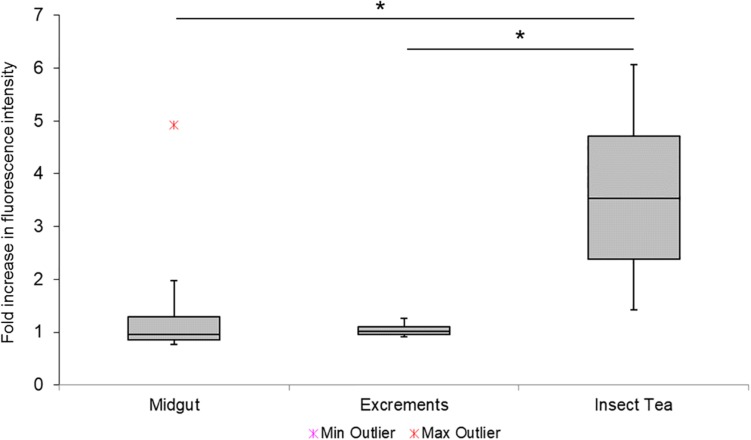
Complementary assessment of *Enterobacteriaceae* during different process steps. The enrichment of *Enterobacteriacea* was confirmed with a complementary qPCR-based approach. The relative fluorescence signal increase in different samples was assessed with group-specific primers, and the fold increase was calculated by using the initial substrate (*C. paliurus* leaves) as an internal reference. Minimum and maximum values are indicated by the whiskers, while outliers are separately shown. Statistical significance (*p* < 0.05) is indicated with a black asterisk.

A qPCR-based approach was used to verify the accumulation of *Enterobacteriaceae* during the production of the insect tea. The results indicated a substantial increase of this taxonomic group in the commercial tea product (Figure 6). It was 3.6-fold more abundant in the insect tea than in the initial substrate (*C. paliurus* leaves). In contrast, neither the midgut nor the excrement samples showed a notable increase in fluorescence intensity when compared to the leaves, indicating a similar prevalence of *Enterobactericeae* in these sample types. Cultivation-dependent assessments have shown that the commercial tea can contain a high number of viable bacteria, which were determined at 2.7 × 10^5^ ± 1.2 × 10^5^ cfu g^–1^. No cell growth was observed on the selective chromogenic coliform agar. The isolation approaches of the morphologically distinct samples have resulted in the detection of *Enterobacter* spp. (seven isolates), *Bordetella* spp. (three isolates), *Bacillus* sp. (three isolates), and *Mixta* sp. (one isolate) as potential contaminants of the commercial tea product. The isolates that were assignable at the species level were identified as *Bordetella petrii* and *Enterobacter hormaechei*.

## Discussion

The findings of the present study show that the insect tea harbors a rich bacterial community that originates from the different steps of the production process. While several health-promoting effects are attested to the insect tea (Xu et al., 2013), it remains unknown which constituents confer these effects. One potential contribution of the insect tea to human health could be the enrichment of the human gut microbiome with the bacteria from this unique beverage similar to fecal transplants that are an efficient treatment for specific inflammatory bowel diseases (Suskind et al., 2015).

We could show that the insect tea harbors a high number of viable bacteria that can be recovered from commercial products. Although only a low number of isolates with distinct morphological features was further analyzed and is therefore not representing the whole cultivable fraction, we found potentially health-relevant bacteria to be present in the insect tea. These microorganisms can be primarily attributed to the digestive system of the tea-producing insect larvae. The occurring members of *Enterobacteriaceae* and *Enterococcaceae* are also considered as classic contaminants in food production (Giraffa, 2002; Takahashi et al., 2017). In the context of the insect tea, it is not clear if the living microorganisms in the final product constitute contaminants or should be considered as active ingredients similar to those in the probiotics or the fermented foods. This is mainly due to the fact that, although the larval excrements are roasted, there is no standardized heat deactivation step involved in the production process that would warranty a contamination-free product. The insect tea and the kopi luwak coffee produced by the Asian palm civet (*Paradoxurus hermaphroditus*) are both unconventional beverages that consist of animal excrements and thus linked to initially high microbial loads. However, the kopi luwak coffee is generally heat-treated at > 200°C (Jumhawan et al., 2013), which is sufficient to deactivate a large proportion of prevalent microorganisms. A study that compared microbial counts in the kopi luwak coffee before and after the beans were roasted showed substantially higher microbial counts before the heat treatment (Marcone, 2004). The counts for aerobic, mesophilic bacteria before the beans were roasted were in the same range as those for the commercial tea product in our study. We therefore assume that a large proportion of the present bacteria survived the roasting process in the insect tea or that the surviving fraction repopulates the product during storage. Although the insect tea is brewed with hot water, preferably at a temperature of 100°C, a high proportion of the content remains at the surface of the water or sticks to the edge of the cup that is used for its preparation. It can be therefore excluded that tea preparation by the final consumers can replace a standardized heat deactivation process that will eliminate all microorganisms.

In order to assess the representativeness of the reconstructed production process, the bacterial compositions of the larvae excrements and of the commercial insect tea were compared. Both sample types showed a similar microbial community composition, although substantial variations in the abundance of distinct groups were evident. These sample types also shared the highest number of common taxonomic groups, indicating a high representativeness of the reconstructed production process. The observed differences might be due to the potential variations of the plant leaves used for insect rearing by the commercial producer. Another important factor is the roasting process that was not included for the laboratory-produced larvae excrements and thus favorable for distinct members of *Proteobacteria*, which were prevalent in the excrements. Various members of this large taxonomic group are generally known to be highly susceptible to heat deactivation (van der Voort et al., 2016). The *Enterobacteriaceae* constitute one of the bacterial signatures of the insect tea; they were enriched during the production process, which was confirmed with complementary analyses. The qPCR-based analyses indicated a substantial enrichment in the product, while the midgut and the excrement samples harbored comparatively lower amounts of quantifiable *Enterobacteriaceae.* We assume that the assessed enterobacterial populations of these samples harbor less-known species that might not be targeted with the implemented primers. While they were identified as a major constituent of all insect-derived samples, they were not identifiable at higher taxonomic resolutions in the 16S rRNA gene fragment datasets nor were they cultivable under the tested conditions. Recent findings related to the plant microbiome and more specifically the leafy green vegetables have shown that the *Enterobacteriaceae* often constitute indigenous members (Rastogi et al., 2013; Erlacher et al., 2014; Cernava et al., 2019b). This makes them a suitable habitat for closely related pathogens that can survive and spread when present (Brandl and Amundson, 2008). However, the direct as well as the indirect implications of the non-pathogenic *Enterobacteriaceae* for human health remain mostly unknown. This led to the natural vaccination hypothesis by Berg et al. (2015) which states that the naturally occurring plant-associated enterobacteria might positively affect our immune system. We have also found that a substantial fraction of the bacterial community present in the final product was assigned to members of *Streptomycetacea*. This bacterial family is primarily known for its antibiotic-producing *Streptomyces* spp. that have been studied for many decades. The highly diverse antibiotic spectrum makes these bacteria very competitive and has resulted in the development of therapeutic antibiotics (Mahajan and Balachandran, 2012) as well as in the common application of these microorganisms for biological control in agriculture (Fravel, 2005). It is likely that the insect tea includes active amounts of bacterial metabolites in addition to its earth-like taste (as assessed by the authors in the frame of conventional tastings) due to the high occurrence of *Streptomycetacea*. In terms of biosafety and human consumption, the *Streptomyces* members rarely cause disease symptoms in humans who are not immunocompromized (Riviere et al., 2012). Therefore, this constituent of the insect tea can be regarded as safe as long as the consumer has a functioning immune system. Another remarkable constituent was identified as *Bordetella petrii* and could be recovered as an isolate from the commercial product that was used in this study. This bacterial species was already found in the gut of related insects (Grabowski and Klein, 2017). While many *Bordetella* species are obligate human pathogens (Weiss and Hewlett, 1986), *B. petrii* was so far primarily isolated from natural environments, although it can also have clinical relevance (Gross et al., 2008). Only a smaller fraction of the identified microorganisms in the product samples originates from the initial substrate (*C. paliurus* leaves). The leaves were shown to harbor a bacterial community that is similar to the phyllosphere microbiomes of the various plant species and especially to those of the perennial plants (e.g., Durand et al., 2018; Cernava et al., 2019a). Although feed is an important factor that shapes the gut microbiome of insects (Lewis and Lizé, 2015), it can be anticipated that only a smaller fraction can adapt to the varying conditions during the formation process of the insect tea. Previous studies focusing on *Drosophila melanogaster* have shown that changes in the diet also result in changes of its gut microbiome (Sharon et al., 2010). We would expect certain effects on the final product when leaves from different plant species are used for insect tea production. However, it remains to be elucidated how pronounced these effects are and if they can be standardized in order to obtain a consistent bacterial community composition. Although *P. farinalis* is a cosmopolitan pest, its gut microbiome was not assessed so far. In the present study we could show that its midgut is mainly colonized by *Firmicutes* and *Proteobacteria*, which corresponds to the findings on other insects (Alonso-Pernas et al., 2017; Kaczmarczyk et al., 2018; van Schooten et al., 2018). While it contributed to the bacterial composition of the insect tea, the final composition is likely affected by handling, processing, and potentially also by storage since not all bacteria are equipped with mechanisms to survive under these conditions, e.g., transition into a dormant stage.

In summary, our microbiome-guided assessment of the bacterial communities in the insect tea has shown that this unconventional beverage contains diverse microbial populations. The reconstruction of the production process and the tracking of bacterial constituents provided a deepening insight into the assemblage of this exotic beverage. The microbial profile is so far unique for an unspoiled food product and contains several bacterial groups that are considered as contaminants in the field of food microbiology. In this context, we could show that a high number of the prevalent microorganisms is viable and can be recovered from the commercial products. The assemblage of the community can be tracked back to specific production steps of the tea but still underlies the dynamics after excretion by the larvae. Although we found a highly diverse bacterial community in the various production steps and recovered a substantial number of living bacteria from a commercial product, deepening analyses of certain taxa would be required to specifically identify human pathogens. While this study confirmed that viable bacteria are a component of insect tea, it remains to be further elucidated if these microbial constituents have any positive or negative implications on human health.

## Data Availability Statement

The datasets generated for this study can be found in the ENA repository (PRJEB32315) and Genbank (MK796111–MK796124).

## Author Contributions

TC and MY conceived the idea and developed the study design. XM and HL performed all laboratory experiments under the supervision of TC and XC. PK performed the general bioinformatics analyses. PK and TC interpreted the bioinformatic data and prepared the final visualizations. GB provided valuable inputs related to microbial ecology and the interpretation of the results. TC, MY, XC, and GB wrote the manuscript. All authors reviewed the final version of the manuscript.

## Conflict of Interest

The authors declare that the research was conducted in the absence of any commercial or financial relationships that could be construed as a potential conflict of interest.

## Footnotes

1 www.earthmicrobiome.org

2 www.ncbi.nlm.nih.gov/nucleotide

3 https://www.ebi.ac.uk/ena

4 https://www.ncbi.nlm.nih.gov/genbank
