# Supplementary material for: Microbiome-Guided Exploration of the Microbial Assemblage of the Exotic Beverage “Insect Tea” Native to Southwestern China
Source: Front Microbiol. 2020 Jan 29;10:3087. doi: 10.3389/fmicb.2019.03087 (PMC7000658; doi:10.3389/fmicb.2019.03087)
Supplement: Supplementary file 1 [file Image_1.pdf]

Supplementary Material

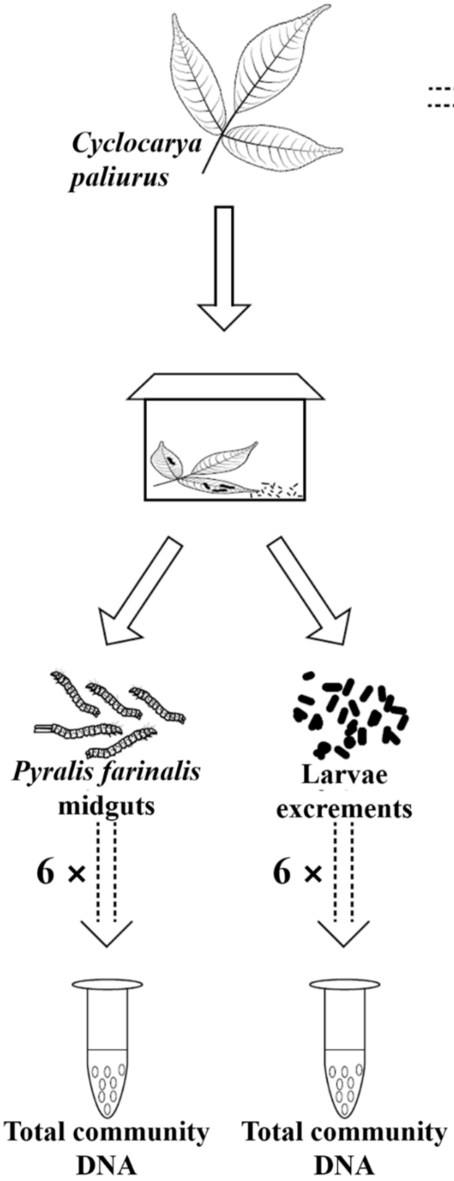

Figure S1. Schematic visualization of sample types and the process workflow. Arrows with solid lines indicate process steps while dashed lines indicate DNA extractions from the pictured sample types. The number of conducted DNA extractions (independent replicates) is shown for each sample type.
